# Salmonellosis-Induced Pericarditis and Pericardial Effusion: A Case Report and Literature Review

**DOI:** 10.7759/cureus.78825

**Published:** 2025-02-10

**Authors:** Talal Aloreibi, Emad Bukhari, Yazeed Terkawi, Mohammed A Miqdad

**Affiliations:** 1 Infectious Diseases, Al Hammadi Hospital, Riyadh, SAU; 2 Cardiac Surgery, Al Hammadi Hospital, Riyadh, SAU; 3 Internal Medicine, Al Hammadi Hospital, Riyadh, SAU; 4 Nephrology, NewYork-Presbyterian, New York, USA

**Keywords:** myopericarditis, non-typhoidal salmonella infections, pericardial effusion, pericardiocentesis, salmonellosis

## Abstract

While salmonellosis is commonly thought to predominantly impact the gastrointestinal system, bacteremia and localized extraintestinal infections such as meningitis, empyema, and pericarditis can develop, particularly in immunocompromised individuals. Here, we present a case of a 69-year-old with multiple comorbidities, who presented to the emergency department with dyspnea and hemodynamics instability in the form of hypoxia and hypotension and was found to have moderate pericardial effusion without echocardiographic signs of tamponade. The ischemic workup was unrevealing, and further infectious workups, including pericardial tissue biopsy and pericardial fluid culture, showed growth in *Salmonella* groups C and D.

Subsequently, the patient was diagnosed with *Salmonella*-induced pericarditis and pericardial effusion and discharged home on oral antibiotics following pericardiocentesis and hemodynamics stabilization. Invasive infections caused by non-typhoidal *Salmonella* are becoming more prevalent in immunocompromised individuals. Acute bacterial myopericarditis is uncommon in advanced nations, yet it can be life-threatening if not treated promptly. Therefore, a high index of suspicion, early detection, and aggressive invasive and non-invasive therapies would strongly be considered to achieve a desirable outcome and good prognosis.

## Introduction

The most prevalent diseases involving the pericardium are pericarditis and pericardial effusion [1]. Acute pericarditis is a potentially fatal disease, and it can be caused by infectious or non-infectious etiologies, such as autoimmune diseases. *Streptococcus pneumoniae*, various *Streptococcus *species, and *Staphylococcus aureus* are common infectious causes of pericarditis [2,3]. While salmonellosis is commonly thought to predominantly impact the gastrointestinal system, bacteremia and localized extraintestinal infections such as meningitis, empyema, and pericarditis may rarely develop, particularly in immunocompromised patients [1]. Invasive infections caused by non-typhoidal *Salmonella* are becoming more prevalent in immunocompromised individuals globally, particularly in impoverished nations [4,5]. Based on a systematic analysis performed in 2017, the highest incidence of non-typhoidal invasive *Salmonella* disease was found in sub-Saharan Africa (34.5 (26.6-45.0) cases per 100000 person-years) and in children younger than five years (34.3 (23.2-54.7) cases per 100000 person-years) [5]. Only a few examples of intrathoracic infection as an extra-intestinal manifestation have been documented in the literature [6].

Myopericarditis is an inflammation of the pericardium and myocardium caused by infection, toxin-mediated diseases, or inflammatory disorders [7,8]. The infectious etiology of acute pericarditis is unknown. Nevertheless, most cases are assumed to be caused by viral infection, either by direct viral cytopathic action or post-viral immune-mediated events [9]. Acute bacterial myopericarditis is uncommon in advanced nations, yet it can be life-threatening if not treated promptly [10]. *Staphylococcus aureus* and *streptococci*, especially *Streptococcus pneumoniae*, are the most prevalent acute bacterial infections. *Neisseria meningitidis*, *Mycoplasma pneumoniae*, and *Campylobacter spp*. are less common causes. *Mycobacterium tuberculosis* is the most prevalent mycobacterial cause of myopericarditis, and it frequently manifests as subacute to chronic symptoms [10].

## Case presentation

**Figure 1:**
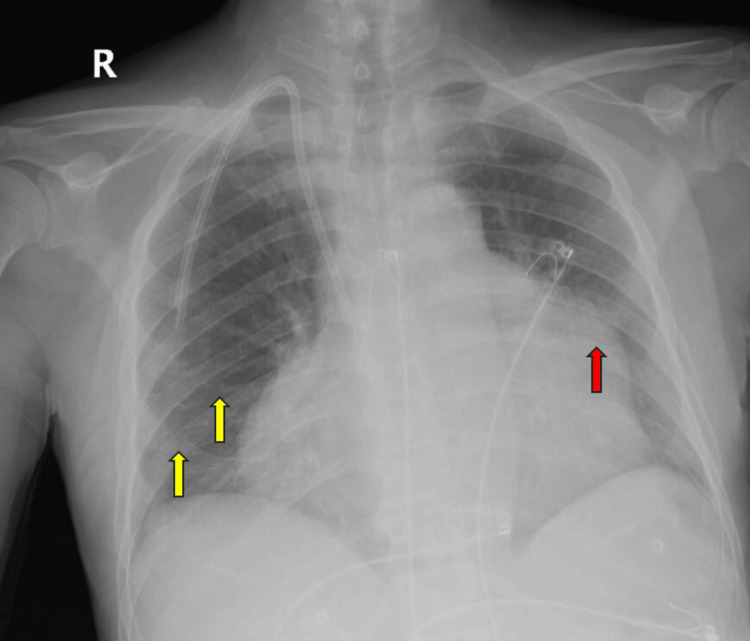
Chest X-ray showing bilateral vascular congestions (yellow arrows) and water-bottle-shaped heart (red arrow).

**Figure 2:**
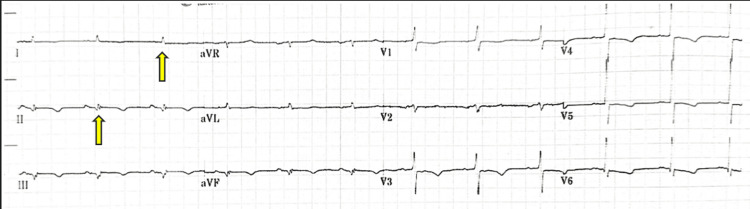
Electrocardiogram showing low QRS voltage (yellow arrows).

A 69-year-old male with a past medical history of type 2 diabetes, hypertension, dyslipidemia, ischemic heart disease with prior coronary artery bypass grafting (CABG), and end-stage renal disease (ESRD) recently started on regular dialysis presented to the emergency department with generalized weakness and shortness of breath for two days duration. He was found to have hypoxia with oxygen saturation (SpO2) of 88% on room air, low blood pressure of 100/60 mmHg, respiratory rate of 24, and temperature of 37.2°C. Physical examinations were remarkable for lower extremities pitting edema, audible bi-basal crackles on lung examination, and congested neck veins. Chest X-ray showed mild interstitial edema (Figure 1). An electrocardiogram (ECG) revealed slightly low QRS voltage, with non-specific ST wave changes (Figure 2). Initial laboratory workup was notable for mild chronic anemia, mild transaminitis, and high inflammatory markers, including white cell counts, C-reactive protein (CRP), and procalcitonin (Table 1). Echocardiogram showed a moderate pericardial effusion mildly compressing the right ventricle. The patient was admitted to the intensive care unit and intubated for worsening work of breath and hypoxia, followed by a hemodialysis session with ultrafiltration for volume optimization. Also, he was started on vasopressors for low blood pressure (86/48 mmHg).

**Table 1:** Laboratory investigations. BUN: blood urea nitrogen; WBC: white blood cells; HGB: hemoglobin; PLT: platelets: INR: international normalized ratio; PT: prothrombin time; CRP: C-reactive protein; ESR: erythrocyte sedimentation rate; ALT: alanine aminotransferase; AST: aspartate aminotransferase.

Labs	Day 1	Day 7	Day 14	Reference range
BUN (mg/dL)	46	55	72	8.9 - 25.7
Calcium (mg/dL)	9.0	9.3	8.2	8.4 - 10.2
Potassium (mmol/L)	5.2	4.8	4.7	3.5 - 5.1
Magnesium (mg/dL)	2.01	2.2	2.1	1.6 - 2.6
Phosphorous (mg/dL)	5.6	5.2	4.5	2.32 - 4.7
Procalcitonin	2.16	10.38	0.74	>2 ng/mL: likely evaluation toward acute sepsis or septic shock
Serum creatinine (mg/dL)	5.35	3.96	4.19	0.72 - 1.25
Sodium (mmol/L)	138	134	133	136 - 145
WBC (x10 ^ 9/L)	7.14	7.32	6.67	4 - 11
HGB (g/dl)	10.9	10.1	9.0	13 - 18
PLT (x10 ^ 9/L)	229	110	226	140 - 450
INR	1.70	1.36	1.25	0.8 - 1.3
PT (seconds)	19.80	15.4	12.9	9.8. - 13.5
CRP (mg/L)	65.20	82.40	28.50	0 - 5
ESR (mm/hour)	22	35	28	0 - 15
Troponin I (ng /ml)	0.074	0.291	0.043	0 - 0.04
ALT (U/L)	891	68	44	0 - 55
AST (U/L)	473.00	56.00	36	5 - 34
Total bilirubin (mg/dL)	0.95	1.32	1.1	0.2 - 1.2
Direct bilirubin (mg/dL)	0.68	0.66	0.4	0 - 0.5
Total protein (g/dl)	6.40	5.40	5.30	6 - 7.8
Serum albumin (g/dl)	3.5	2.8	3.2	3.2 - 4.5

It was initially thought that the presenting symptoms were secondary to ischemic heart disease, given multiple comorbidities and high cardiovascular risks. Hence, a diagnostic coronary angiography was performed and revealed severe left anterior descending artery calcification with proximal mild and distal tandem lesions of 80%, and left circumflex had diffuse severe calcification of 70% stenosis at the first obtuse marginal artery of 80% at mid-segment and right posterior descending artery. A subsequent echocardiogram showed worsening pericardial effusion with a possible component of hematoma. Accordingly, a decision was made by a multidisciplinary team, including a cardiac surgeon, cardiologist, nephrologist, and an intensivist, for urgent open-heart surgery to release the tamponade with CABG and intra-aortic balloon pump. The patient underwent two coronary artery bypass grafts (left anterior descending artery, right coronary artery) in addition to pericardiocentesis with drainage of pericardial effusion. Furthermore, two intercostal drain tubes were inserted, and intravenous meropenem (1 gm/day) and vancomycin (1 gm post dialysis) were started empirically until *Salmonella* groups C and D were grown in pericardial fluid culture, which was susceptible to meropenem. Pericardial tissue biopsy showed central areas of fibrinoid necrosis and active granulation tissue densely infiltrated mostly by neutrophils and a few mononuclear cells. Foci of patchy fibrosis and areas of myofibroblastic proliferation were also seen.

As a result, the patient’s clinical status improved following CABG and pericardial fluid drainage, while maintained on intravenous meropenem alone. The patient required less O2 and vasopressors, and inflammatory markers started to trend down slowly (Table 1). On postoperative day 11, the patient was extubated and then downgraded to the medical floor with normal hemodynamics. A follow-up echocardiogram showed trace pericardial effusion. He was discharged home on oral antibiotics: oral ciprofloxacin 500 mg twice daily, oral linezolid 600 mg twice daily, and oral augmentin 1000 mg twice daily, all for five days, with close follow-up in the infectious disease and cardiology clinics. The patient showed up one week later in the clinic. He was in great condition with no new symptoms and reported significant respiratory improvement and overall physical functionality. The echocardiogram was not repeated, given the clinical improvement without acute symptoms. Routine comprehensive metabolic panel, CRP, and blood count tests were unremarkable from the infection point of view.

## Discussion

Non-typhoidal *Salmonella* are gram-negative bacteria that often induce self-limiting gastroenteritis but can also cause invasive illness. Non-typhoidal *Salmonella* is predicted to kill 90,300 people per year, and antibiotic resistance is becoming an increasing concern [11]. It is the second most prevalent food-related infection in Europe, mainly caught by consumption of infected meat, eggs, or milk [12]. *Salmonella* infection is generally severe and affects the gastrointestinal tract. Extraintestinal non-typhoidal *Salmonella* infection is less common than intestinal non-typhoidal *Salmonella* infection [13]. Non-typhoidal *Salmonella* infection beyond the gastrointestinal tract is frequent in the meninges, musculoskeletal system, and urinary tract. Pericardial involvement, on the other hand, is believed to be 2% [14]. Chehab et al. reported a case of Group B *Salmonella meningitis* and 10 cases of *S. enteritidis* myopericarditis published in the literature, 70% of whom were immunocompromised, primarily due to long-term corticosteroid usage. Of the 10 instances, 70% had tamponade, 30% had constrictive pericarditis, 40% had pericardiectomy, and 20% died [7].

Two types of risk factors might lead to intrathoracic non-typhoidal *Salmonella* infections: systemic and local [4]. HIV, malaria, diabetes, uremia, poor cell-mediated immunity, poor B-cell function, past antimicrobial usage, decreased stomach acidity, and low socioeconomic level are examples of systemic variables. Prior lung or pleural illness, as well as congenital anomalies, are examples of local causes [15,16]. ESRD is not classified as an immunocompromised condition. Yet, in individuals with impaired renal function, both innate and adaptive immunity are disrupted by the inflammatory uremic milieu [17]. ESRD patients have reduced neutrophil-killing capability, and the functional impairment described in uremic neutrophils is primarily due to their reduced ability to kill microorganisms intracellularly, which is thought to increase vulnerability to infections. Inflammations caused by hemodialysis exacerbate the weakened immune system [18]. These immune system disruptions might explain why some dialysis patients acquired a non-typhoidal *Salmonella* lung abscess in the absence of established risk factors linked with salmonellosis-related extraintestinal illness [19]. As in our case, ESRD and diabetes may impair the patient's immune response, making him more susceptible to extraintestinal salmonellosis.

Jin et al. identified 42 recorded cases of non-typhoidal *Salmonella* in a review of the literature, with nine cases of myocarditis, six cases of myopericarditis, and 27 cases of pericarditis. Overall, *Salmonella enteritidis* was found to be the most frequent organism, accounting for 42.9% of all reported cases, followed by *Salmonella typhimurium* in 31.7%. Tamponade was the most usually reported complication, particularly in pericarditis, whereas ventricular rupture was the most documented complication of myocarditis [19].

Myocarditis often manifests as chest pain, often of varying severity, and it can be difficult to distinguish it from ischemic chest pain. Otherwise, the presenting symptoms might be heart failure, flu-like symptoms, tiredness, palpitations, syncope, or even sudden cardiac death. Pericarditis is characterized by pleuritic chest discomfort, which is typically eased by leaning forward, as well as weariness, palpitations, dyspnea, and in certain cases, tamponade [20]. The pericardial friction rub is a typical exam finding, commonly characterized as a scraping sound best heard above the left sternal border. Purulent pericarditis is a rare subgroup in the antimicrobial age, but it should be evaluated because of its significant mortality and morbidity. Symptoms of pericarditis may be present, especially if coupled with sepsis or bacteremia, with *S. aureus* being the most usually diagnosed bacteria. ECG, echocardiography, laboratory blood tests, biopsies, coronary angiography, and cardiac magnetic resonance imaging are all relevant investigations in myopericarditis. ECG alterations vary but often comprise broad ST elevation and PR depression, followed by segment normalization and T-wave inversion [21].

Another important area of inquiry is echocardiography, which may detect ventricular dysfunction, valve incompetence, and thrombus in myocarditis, as well as the level of pericardial involvement, which can range from entirely normal to cardiac tamponade in pericarditis [22]. Cardiac magnetic resonance imaging is another potentially useful inquiry since it may properly detect inflammation in either the pericardium or the myocardium and assist in differentiating between acute coronary syndrome [10,23].

Cardiac tamponade is an emergency that demands early identification and recognition based on clinical signs and is validated by echocardiography. In individuals with cardiac tamponade, urgent intervention is required to elevate the malfunction of the right ventricle [24,25]. Purulent pericarditis should be treated as soon as possible, with drainage of contaminated fluid and empiric broad-spectrum antibiotics. Gram, acid-fast, fungal stains, and microbiological cultures should be used to examine the pericardial fluid. Antibiotic medication can then be modified based on culture and sensitivity findings. Percutaneous pericardiocentesis is a straightforward and safe procedure for removing bacterial infections [26]. However, in situations with loculations with fibrin buildup and severe adhesions, percutaneous drainage may be insufficient and inadequate, necessitating a pericardial window or extensive pericardiectomy. The European Society of Cardiology recommends open surgical drainage as the preferred treatment [27]. Furthermore, intrapericardial fibrinolysis using urokinase has been used to prevent recurrence and reduce the risk of constrictive pericarditis [28]. In our case, we decided to do open heart surgery, aiming for diagnostic/therapeutic pericardiocentesis and CABG simultaneously.

Despite intensive therapy, purulent pericarditis has a high risk of progressing to constrictive pericarditis. In this case, significant pericardiectomy is not necessary to release the pericardial restriction. Although the majority of patients finally had the thorough pericardiectomy, the prognosis is positive in those who receive prompt treatment. Because *Salmonella* empyema has a high death rate, it is critical to have early detection and diagnostic investigation. A greater rate of effective recovery is related to aggressive intervention, which involves immediate pericardiotomy and early start of suitable antibiotics [29-31]. In our case, early detection and aggressive invasive intervention with broad-spectrum antimicrobial coverage have fortunately prompted a good outcome and prognosis.

## Conclusions

Invasive infections caused by non-typhoidal *Salmonella* are becoming more prevalent in immunocompromised individuals, especially with diabetic patients and patients with ESRD who require regular dialysis. The majority of myopericarditis cases, however, are assumed to be caused by viral infection, either by direct viral cytopathic action or through post-viral immune-mediated events. Acute bacterial myopericarditis is uncommon in advanced nations, yet it can be life-threatening if not treated promptly. This case highlights the importance of considering non-typhoidal *Salmonella *as a rare but serious cause of pericarditis, particularly in immunocompromised patients. Therefore, early detection, aggressive invasive and non-invasive interventions, and targeted antibiotic therapy are crucial for recovery.
